# Exosomal microRNAs in the DLK1-DIO3 imprinted region derived from cancer-associated fibroblasts promote progression of hepatocellular carcinoma by targeting hedgehog interacting protein

**DOI:** 10.1186/s12876-022-02594-2

**Published:** 2022-12-08

**Authors:** An-Li Jin, Lin Ding, Wen-Jing Yang, Te Liu, Wei Chen, Tong Li, Chun-Yan Zhang, Bai-Shen Pan, Shuang-Jian Qiu, Jian Zhou, Jia Fan, Wei Guo, Xin-Rong Yang, Bei-Li Wang

**Affiliations:** 1 Department of Laboratory Medicine, Zhongshan Hospital, Fudan University, Shanghai, 200032 People’s Republic of China; 2 Department of Liver Surgery and Transplantation, Liver Cancer Institute, Zhongshan Hospital, Fudan University, Shanghai, 200032 People’s Republic of China; 3 Key Laboratory of Carcinogenesis and Cancer Invasion, Ministry of Education, Shanghai, 200032 People’s Republic of China; 4 Department of Laboratory Medicine, Xiamen Branch, Zhongshan Hospital, Fudan University, Xiamen, 361015 People’s Republic of China; 5 Department of Laboratory Medicine, Wusong Branch, Zhongshan Hospital, Fudan University, Shanghai, 200940 People’s Republic of China; 6 Cancer Center, Zhongshan Hospital, Fudan University, Shanghai, 200032 People’s Republic of China; 7 Shanghai Geriatric Institute of Chinese Medicine, Shanghai University of Traditional Chinese Medicine, Shanghai, 200031 People’s Republic of China; 8 Branch of National Clinical Research Center for Laboratory Medicine, Shanghai, 200031 People’s Republic of China

**Keywords:** Hepatocellular carcinoma, Cancer-associated fibroblasts, Exosomes, DLK1-DIO3 microRNA cluster, Hedgehog interacting protein

## Abstract

**Background:**

Hepatocellular carcinoma (HCC) is the sixth most commonly diagnosed cancer and third leading cause of cancer-related death worldwide in 2020. Exosomes derived from cancer-associated fibroblasts (CAFs-exo) can promote tumor progression in various human cancers. However, the underlying regulatory mechanism controlling how CAFs-exo can promote HCC progression remains poorly understood.

**Methods:**

CAFs and para-cancer fibroblasts (PAFs) were isolated from HCC tissues and corresponding para-cancer tissues, then were cultured in vitro. CAFs and PAFs were characterized by immunofluorescence and western blot (WB) assays. Exosomes were isolated by ultracentrifugation, and characterized by transmission electron microscopy, nanoflow cytometry, and WB assay. The internalization of exosomes by HCC cells was observed under a fluorescence microscope. Cell Counting Kit-8 (CCK-8) assay was used to evaluate cell proliferation. Wound healing and transwell assays were used for migration and invasion experiments. RT-PCR assay was used to examine differentially expressed microRNAs (miRNAs) in exosomes and HCC cells. The TargetScan database was used to predict miRNA target genes. Hedgehog interacting protein (HHIP) expression analysis, prognostic analysis, and enrichment analysis of HHIP-related co-expressed genes were performed using the TIMER, UALCAN, Kaplan–Meier plotter, and LinkedOmics databases.

**Results:**

CAFs-exo were internalized by HCC cells. CAFs-exo contributed to the aggressive phenotype of HCC cells, while inhibiting exosome secretion reversed these effects. Mechanistically, miRNAs in the DLK1-DIO3 imprinted region (miR-329-3p, miR-380-3p, miR-410-5p, miR-431-5p) were increased in HCC cells co-cultured with CAFs-exo compared with PAFs-exo. Expression of HHIP, a possible miR-431-5p target gene, was significantly downregulated in HCC cells. Low HHIP expression level in tumor tissues could predict poor prognosis in HCC patients. HHIP-related co-expressed genes were mainly associated with cell adhesion molecules.

**Conclusions:**

CAFs-exo can promote HCC progression by delivering miRNAs in the DLK1-DIO3 imprinted region to HCC cells, subsequently inhibiting HHIP expression. HHIP is a potential prognostic biomarker in HCC.

**Supplementary Information:**

The online version contains supplementary material available at 10.1186/s12876-022-02594-2.

## Background

Hepatocellular carcinoma (HCC) accounts for 75% to 85% of primary liver cancer cases, which ranks as the sixth most commonly diagnosed cancer type and the third leading cause of cancer-related death worldwide in 2020 [1]. Although surgical resection remains the most effective HCC treatment method, only 20% to 30% of HCC patients have the opportunity for surgery because many are already at an advanced stage when they are diagnosed [2]. The underlying mechanisms of HCC progression are not fully understood. Therefore, it is critical to explore these mechanisms to support the further development of novel therapeutic strategies for HCC treatment.

The tumor microenvironment (TME) is considered a major contributor to tumor progression [3]. The TME is composed of tumor cells, innate and adaptive immune cells, cancer-associated fibroblasts (CAFs), and other cell types [4]. Several studies found that CAFs promoted tumor progression through their interaction with tumor cells [5, 6]. Because of the important role of CAFs in the TME, they are potential therapeutic targets for anti-cancer therapies [7]. Exosomes are cell-derived nanovesicles containing cargo that can include nucleic acids, proteins, and lipids. Exosomes function as major mediators of intercellular communication by transferring various genetic information within the cargo when secreted by one cell and taken up by another [8]. Exosomes derived from CAFs (CAFs-exo) could reportedly promote tumor progression in various human cancers [9, 10], suggesting that CAFs-exo played a significant role in the TME. However, the mechanisms by which CAFs-exo can promote HCC progression and details of the communication between CAFs-exo and HCC cells are still unclear.

MicroRNAs (miRNAs) are a class of small endogenous non-coding RNAs that are typically between 19 and 23 nucleotides (nt) in length. MiRNAs inhibit gene expression post-transcriptionally by binding to the complementary 3′ untranslated region (3′-UTR) of the mRNA of the target gene, leading to mRNA cleavage or translational inhibition [11]. Increasing evidences showed that miRNAs were frequently abnormally expressed in tumor tissues, suggesting that these molecules had important roles in tumor progression and metastasis [12]. The DLK1-DIO3 imprinted region is located on human chromosome 14 and mouse chromosome 12, containing the paternally expressed genes delta-like homolog 1 (DLK1), retrotransposon-like gene 1 (RTL1), and type 3 deiodinase (DIO3), and the maternally expressed genes maternally expressed gene 3 (MEG3), MEG8, and antisense RTL1 [13]. In addition, the DLK1-DIO3 imprinted region contains 54 miRNAs, which represents one of the largest miRNA clusters in the human genome [14]. The miRNAs in the DLK1-DIO3 imprinted region are involved in the epithelial-mesenchymal transition (EMT) process and oncogenesis [15, 16]. However, the specific regulatory mechanisms of these miRNAs in the DLK1-DIO3 imprinted region in HCC cells remain unknown.

In this study, we explored the role and mechanisms of CAFs-exo in promoting HCC progression. Our data suggested that CAFs-exo could be internalized by HCC cells, delivering miRNAs in the DLK1-DIO3 imprinted region, and thus, promoting the aggressive phenotype of HCC cells. In vitro experiments showed that the expression of hedgehog interacting protein (HHIP) was inhibited in HCC cells co-cultured with CAFs-exo. Bioinformatics analysis further indicated that HHIP expression level was downregulated in HCC tissues, and low HHIP expression could predict poor prognosis in HCC patients. Our findings provide novel insights into HCC progression, which will contribute to the development of novel therapeutics for HCC treatment.

## Methods and materials

### Clinical specimens

Clinical samples were collected from HCC patients undergoing surgical resection in the Liver Cancer Institute, Zhongshan Hospital, Fudan University. HCC patients were diagnosed by hematoxylin–eosin staining without a history of radiotherapy or chemotherapy before surgical resection. Approval for the use of human specimens was obtained from the Research Ethics Committee of Zhongshan Hospital, Fudan University. Informed consent had been obtained from all patients.

### Cell culture and transfection

HCC cell lines (PLC/PRF/5 and SMMC7721) were obtained from the Chinese Academy of Sciences (Shanghai, China). HCC cells were cultured in high-glucose Dulbecco’s modified Eagle’s medium (DMEM, Gibco BRL, Grand Island, NY) supplemented with 10% fetal bovine serum (FBS, Gibco BRL), 1% penicillin and 100 μg/ml streptomycin (Gibco BRL) at 37 °C with 5% CO_2_. MiRNA mimics were designed and synthesized by GenePharma (Shanghai, China), and were transfected into HCC cells using a Lipo3000 kit (Invitrogen, Carlsbad, CA, USA) according to manufacturer’s instructions.

### Isolation and culture of CAFs and para-cancer fibroblasts (PAFs)

CAFs and PAFs were isolated from HCC tissues and corresponding para-cancer tissues respectively. Tissues were minced with a sterile blade into pieces (1 mm^3^) and were digested for 2 h at 37 °C in 10% FBS DMEM containing 1 mg/mL collagenase type IV (Sigma-Aldrich, Atlanta, GA, USA). Then samples were filtered through an 8 μm mesh to remove undigested debris and were cultured in DMEM/F12 (Gibco BRL) supplemented with 10% FBS, 1% penicillin and 100 μg/ml streptomycin at 37 °C with 5% CO_2_. Non-adherent cells were removed by washing with PBS after 48 h. The adherent fibroblasts were incubated in a 24-well plate, and then transferred to a 25cm^3^ cell culture flask for cell expansion. Primary fibroblasts were used for experiments up to passage 10.

### Immunofluorescence (IF) assay

Cells were fixed in 4% paraformaldehyde and blocked with 5% bovine serum albumin. Next, cells were incubated with anti-α-SMA (1:100, Cell Signaling Technology, CST, USA) and anti-Vimentin (1:100, CST) at 4 °C for a night, followed by incubation with 488-conjugated secondary antibody (CST) for 2 h. After washing with PBS, cells were counterstained with DAPI for observation under the microscope. Average optical density (AOD) was used for the quantification of the results of IF assay. AOD = integrated option density (IOD) / Area.

### Western blot (WB)

WB assay was carried out using standard procedures as previously descried [17]. The antibodies used for WB assay were as follows: anti-α-SMA (CST), anti-Vimentin (CST), TSG101 (Abcam, Cambridge, MA), CD81 (CST), CD9 (CST), E-cadherin (CST), N-cadherin (CST), HHIP (Affinity Biosciences, Changzhou, China), and GAPDH (Beyotime, Shanghai, China). The protein level was standardized to GAPDH, and then standardized to experimental control. Densitometric analysis was performed using NIH Image J software.

### Isolation of exosomes

The fibroblasts were incubated with 10% FBS DMEM/F12 for 48 h. The culture medium was collected, and then centrifuged at 1,000 × g for 5 min and 10,000 × g for 30 min to remove cell debris, followed by filtration through a 0.22 μm filter (Millipore, USA). Exosomes were pelleted by ultracentrifugation at 120,000 × g for 70 min for further in vitro experiments.

### Characterization of exosomes

The morphology of exosomes was observed by the transmission electron microscopy analysis. The size distribution of exosomes was analyzed by the nanoflow cytometry analysis. The procedure of transmission electron microscopy analysis and nanoflow cytometry analysis was the same as our previous study [18].

### Exosomes internalization assay

CAFs were labeled with fluorescent dye 1,1'-dioctadecyl-3,3,3',3'-tetramethylindocarbocyanine perchlorate (Dil, Thermo Scientific, USA), and incubated for 20 min at 37 °C. HCC cells were plated in a 24-well plate, followed by incubation with Dil-labeled CAFs for 24 h at 37 °C in the upper transwell chamber for observation under a fluorescence microscope.

### Cell Counting Kit-8 (CCK-8), wound healing, and transwell assays

For CCK-8 assay, cells (2000 cells per well) were incubated in a 96-well plate. Cell viability were examined at 0 day, 1 day, 2 day, 3 day and 4 day. All operations were carried out according to manufacturer’s instructions. The abilities of cell migration and invasion were detected by wound healing assay and transwell assay as previously described [17]. All experiments were conducted in triplicate.

### RNA extraction and RT-PCR

Total RNA of cells was extracted by the RNA isolation kits (Qiagen, Germany). For mRNA detection, cDNA was synthesized by the Quantitect reverse transcription kit (Qiagen). All operations were carried out according to manufacturer’s instructions. The mRNA expression levels of genes were quantified by SYBR Mix (Takara, Japan) and Roche real-time PCR detection system (Roche Diagnostics). The primers used in this study were as follows: KFL12: 5′-CCTCACCTTCTTCAACTTCAAC-3′(F), 5′-GCCTCCAACACCAGATGC-3′(R); KDM5A: 5′-AGGATAGGAAATACCCAGAGAATG-3′(F), 5′-GAGCCACAGAAGCACAGG-3′(R); HHIP: 5′-GTGCTACGGCTGGATGTG-3′(F), 5′-TGGTGCTGTTGAAGTGTGG-3′(R); GAPDH: 5′-CCACTCCTCCACCTTTGAC-3′(F), 5′-CACCACCCTGTTGCTGTAG-3′(R). PCR conditions were as follows: 5 min at 95 °C, followed by 40 cycles of 95 °C for 10 s and 60 °C for 60 s. GAPDH was used as an internal control. For miRNAs detection, cDNA synthesis and quantitative RT-PCR detection were conducted by miRNA qRT-PCR detection kit (GeneCopoeia, Rockville, Maryland). The primers of miRNAs used in this study were designed and synthesized by GeneCopoeia. PCR conditions were as follows: 5 min at 95 °C, followed by 40 cycles of 95 °C for 10 s, 60 °C for 20 s, and 72 °C for 10 s. U6 was used as an internal control. The fold change was calculated according to the formula 2^−ΔΔCt^. Each reaction was performed in triplicate.

### Bioinformatics analysis

The target genes of miRNAs were predicted by the TargetScan database (http://www.targetscan.org/vert_72/). The venn diagram was plotted by the VENNY 2.1 (https://bioinfogp.cnb.csic.es/tools/venny/) [19]. The analysis of HHIP expression in human cancers was performed by the TIMER database (https://cistrome.shinyapps.io/timer/) [20]. The analysis of the differential expression of HHIP was performed in liver hepatocellular carcinoma (LIHC) cohort from The Cancer Genome Atlas (TCGA) database (https://portal.gdc.cancer.gov), HCC cohorts from the Gene Expression Omnibus (GEO) database (https://www.ncbi.nlm.nih.gov/), and Liver Cancer-RIKEN, JP (LIRI-JP) cohort from the International Cancer Genome Consortium (ICGC) data portal (https://dcc.icgc.org/). The Kaplan–Meier (KM) survival curves were plotted using the KM plotter database (http://kmplot.com/analysis/index.php?p=service&cancer=liver_rnaseq) [21]. The analysis of the HHIP-related co-expressed genes in TCGA-LIHC cohort was performed using the LinkedOmics database (http://www.linkedomics.org/) [22]. Enrichment analysis was performed using the LinkInterpreter module of LinkedOmics. Gene set enrichment analysis (GSEA) tool was used to perform the enrichment analysis, including Gene Ontology (GO) terms biological processes (BP), cellular components (CC), and molecular functions (MF), and Kyoto Encyclopedia of Genes and Genomes (KEGG) pathways [23–25].

### Statistical analysis

SPSS software 16.0 and Graphpad prism 8.0 were used for statistical analysis. The χ^2^ test, Fisher’s exact test, Student’s *t* test, and Mann–Whitney *U* test were used to evaluate the significance of differences in data between groups. *p* < 0.05 was considered statistically significant.

## Results

### Isolation and identification of CAFs and CAFs-derived exosomes

**Fig. 1:**
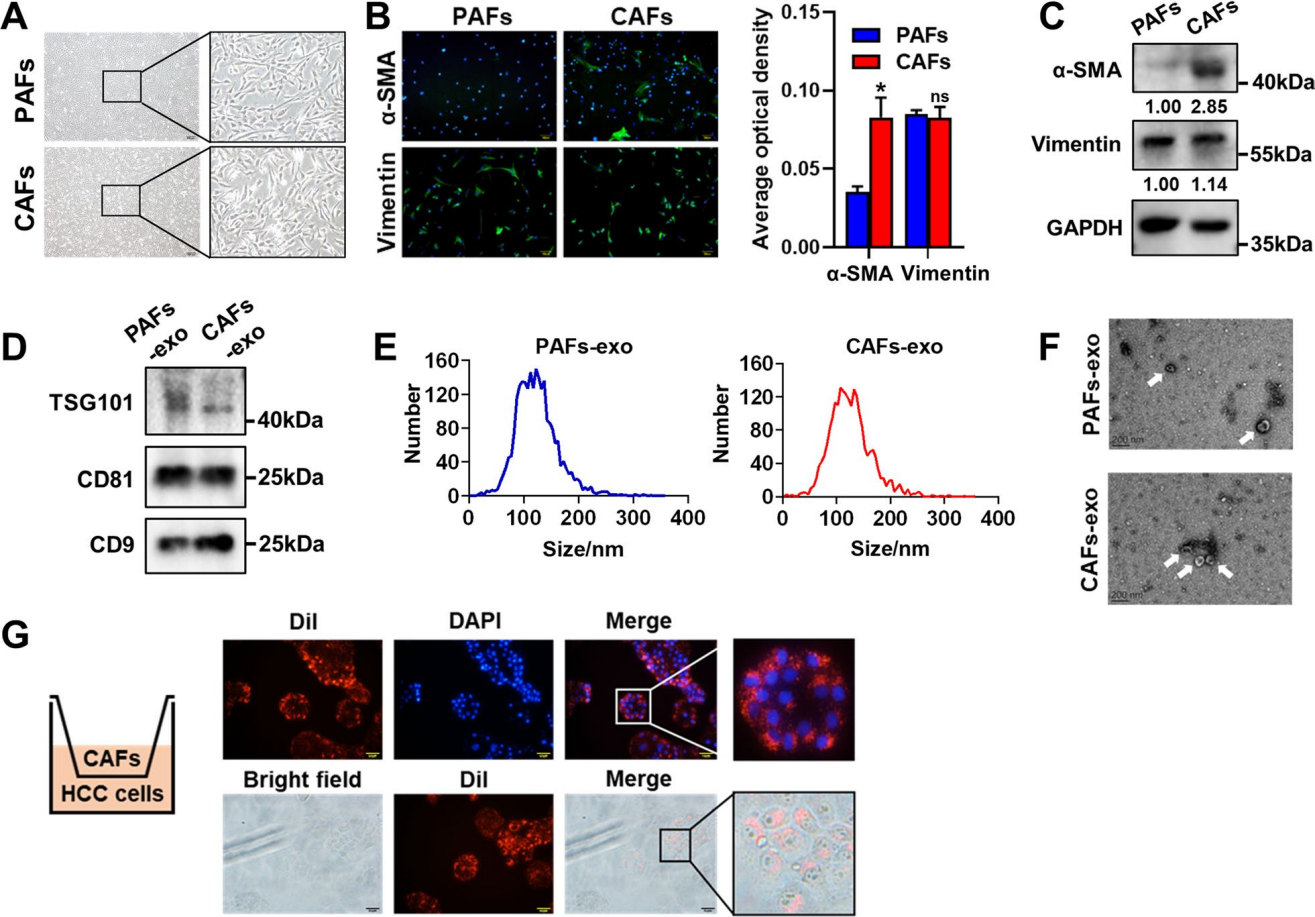
Characteristics of cancer-associated fibroblasts (CAFs) and CAFs-derived exosomes (CAFs-exo). **A** Representative morphology of CAFs and para-cancer fibroblasts (PAFs) isolated from hepatocellular carcinoma (HCC) patients. Scale bar: 200 μm. **B** Representative immunofluorescence (IF) images of α-SMA and Vimentin in CAFs and PAFs. Scale bar: 100 μm. **C** The protein expression levels of α-SMA and Vimentin in CAFs and PAFs were detected by western blot (WB) assay. **D** The protein expression levels of TSG101, CD81, and CD9 in CAFs-exo and PAFs-exo were detected by WB assay. **E** Size distribution of CAFs-exo and PAFs-exo was analyzed by nanoflow cytometry analysis. **F** Micrograph of CAFs-exo and PAFs-exo was visualized by transmission electron microscopy analysis. Scale bar: 200 nm. **G** Representative IF images of PLC/PRF/5 cells co-cultured with Dil-labelled CAFs. Scale bar: 50 μm

We first characterized a spindle-shaped morphology of PAFs and CAFs (Fig. 1A). IF and WB assays showed that both PAFs and CAFs expressed the mesenchymal marker (Vimentin), and the expression level of CAF-specific marker (α-SMA) was higher in CAFs compared with PAFs (Fig. 1B, C; Additional file 1: Fig. S1). We further isolated exosomes from the conditioned medium of CAFs (CAFs-CM) and PAFs (PAFs-CM) using ultracentrifugation. WB assay showed that the classic exosomal markers (TSG101, CD81, CD9) were expressed in both PAFs-derived exosomes (PAFs-exo) and CAFs-derived exosomes (CAFs-exo) (Fig. 1D; Additional file 1: Fig. S2). Transmission electron microscopy and nanoflow cytometry analysis also confirmed the typical cup-shaped morphology of exosomes, which ranged in size from 50 to 200 nm (Fig. 1E, F).

To determine whether CAFs-exo could be internalized by HCC cells, CAFs were labeled with fluorescent dye Dil, and were then incubated with HCC cells in the upper transwell chamber for 24 h. We found that red fluorescence signal was observed in HCC cells in the lower transwell chamber (Fig. 1G).

### CAFs-exo can promote the aggressive phenotype of HCC cells

**Fig. 2:**
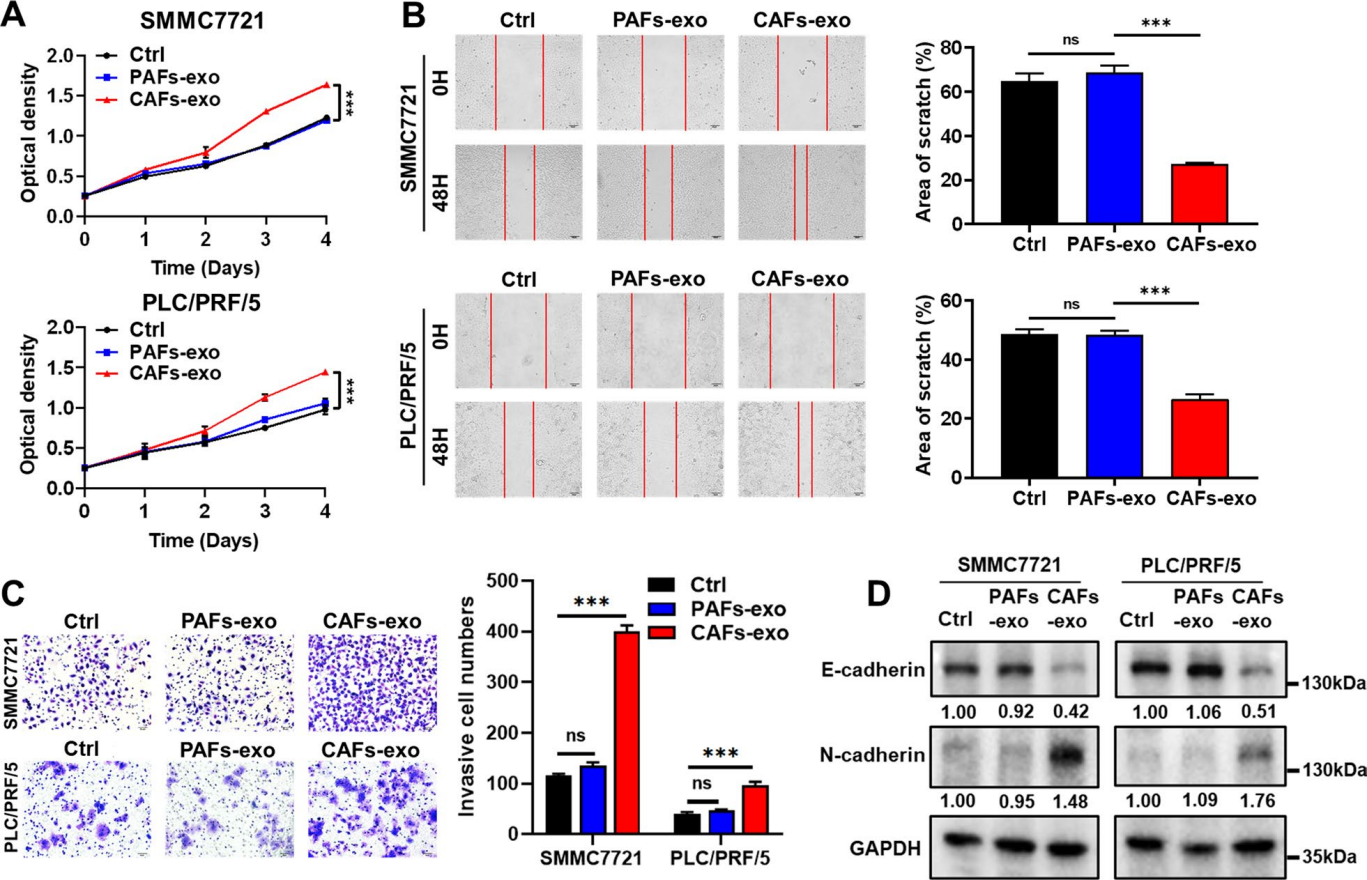
CAFs-exo promote proliferation, migration, and invasion in HCC cells. **A** The effects of PAFs-exo and CAFs-exo on HCC cell proliferation were evaluated by CCK-8 assay. **B** The effects of PAFs-exo and CAFs-exo on HCC cell migration were evaluated by wound healing assay. Scale bar: 100 μm. **C** The effects of PAFs-exo and CAFs-exo on HCC cell invasion were evaluated by transwell assay. Scale bar: 50 μm. **D** The protein expression levels of epithelial-mesenchymal transition (EMT)-related markers in HCC cells treated with PAFs-exo and CAFs-exo were detected by WB assay

**Fig. 3:**
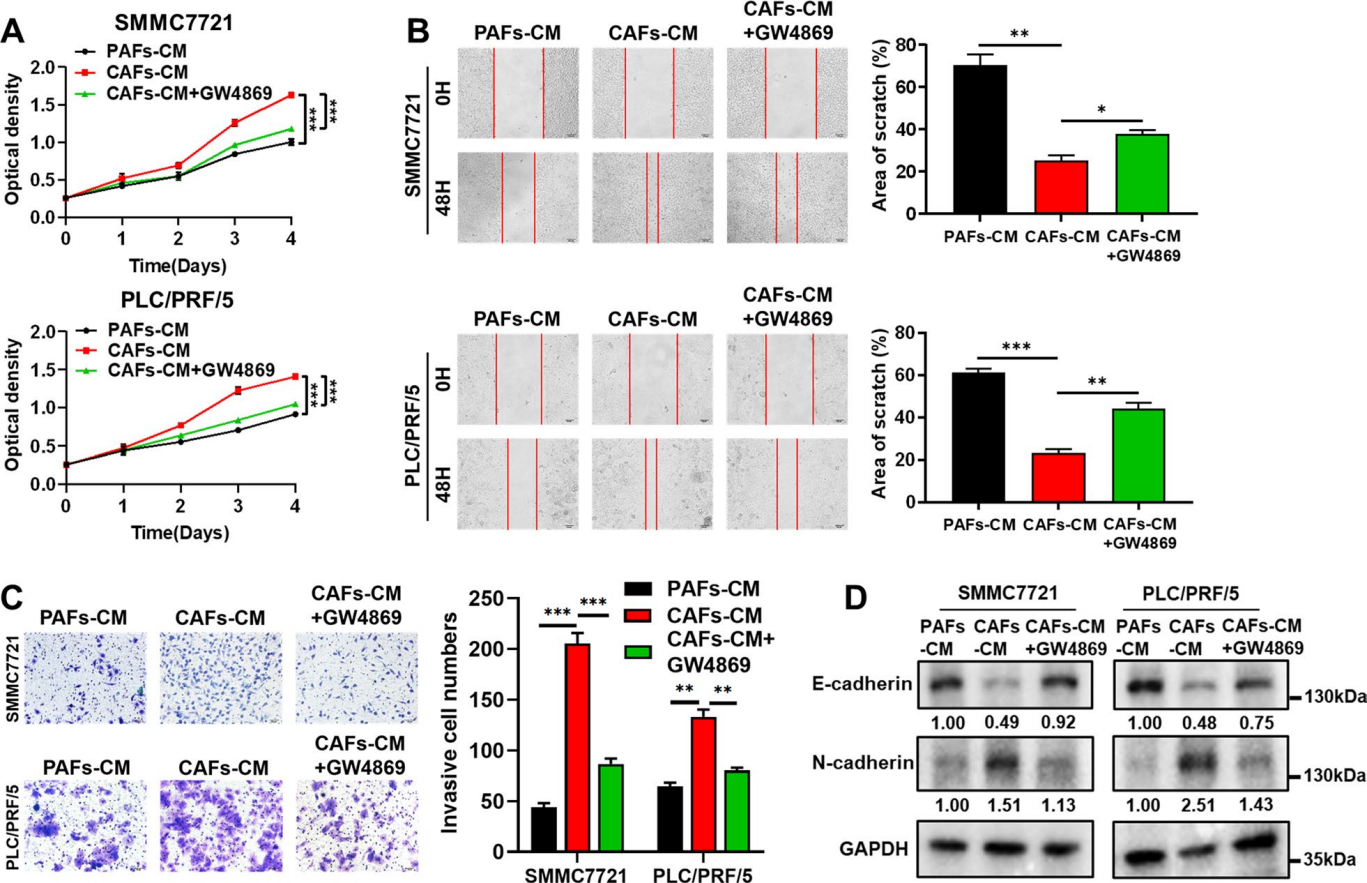
Inhibition of exosome secretion suppresses the aggressive phenotype of HCC cells. **A** The effects of conditioned medium of PAFs (PAFs-CM), CAFs-CM, and CAFs-CM + GW4869 on HCC cell proliferation were evaluated by CCK-8 assay. **B** The effects of PAFs-CM, CAFs-CM, and CAFs-CM + GW4869 on HCC cell migration were evaluated by wound healing assay. Scale bar: 100 μm. **C** The effects of PAFs-CM, CAFs-CM, and CAFs-CM + GW4869 on HCC cell invasion were evaluated by transwell assay. Scale bar: 50 μm. **D** The protein expression levels of EMT-related markers in HCC cells treated with PAFs-CM, CAFs-CM, and CAFs-CM + GW4869 were detected by WB assay

CCK-8 assay showed that CAFs-exo could promote cell proliferation compared with PAFs-exo (Fig. 2A). Wound healing and transwell assays demonstrated that CAFs-exo could enhance the migration and invasion rates in HCC cells compared with PAFs-exo (Fig. 2B, C). Moreover, co-cultures with CAFs-exo resulted in lower E-cadherin expression and higher N-cadherin expression in HCC cells (Fig. 2D; Additional file 1: Fig. S3). GW4869 treatment was used to inhibit exosome secretion from CAFs, which reversed the observed effects in HCC cells (Fig. 3A–D; Additional file 1: Fig. S3).

### HHIP expression is inhibited by the CAFs-exo-derived miRNAs in the DLK1-DIO3 imprinted region

**Fig. 4:**
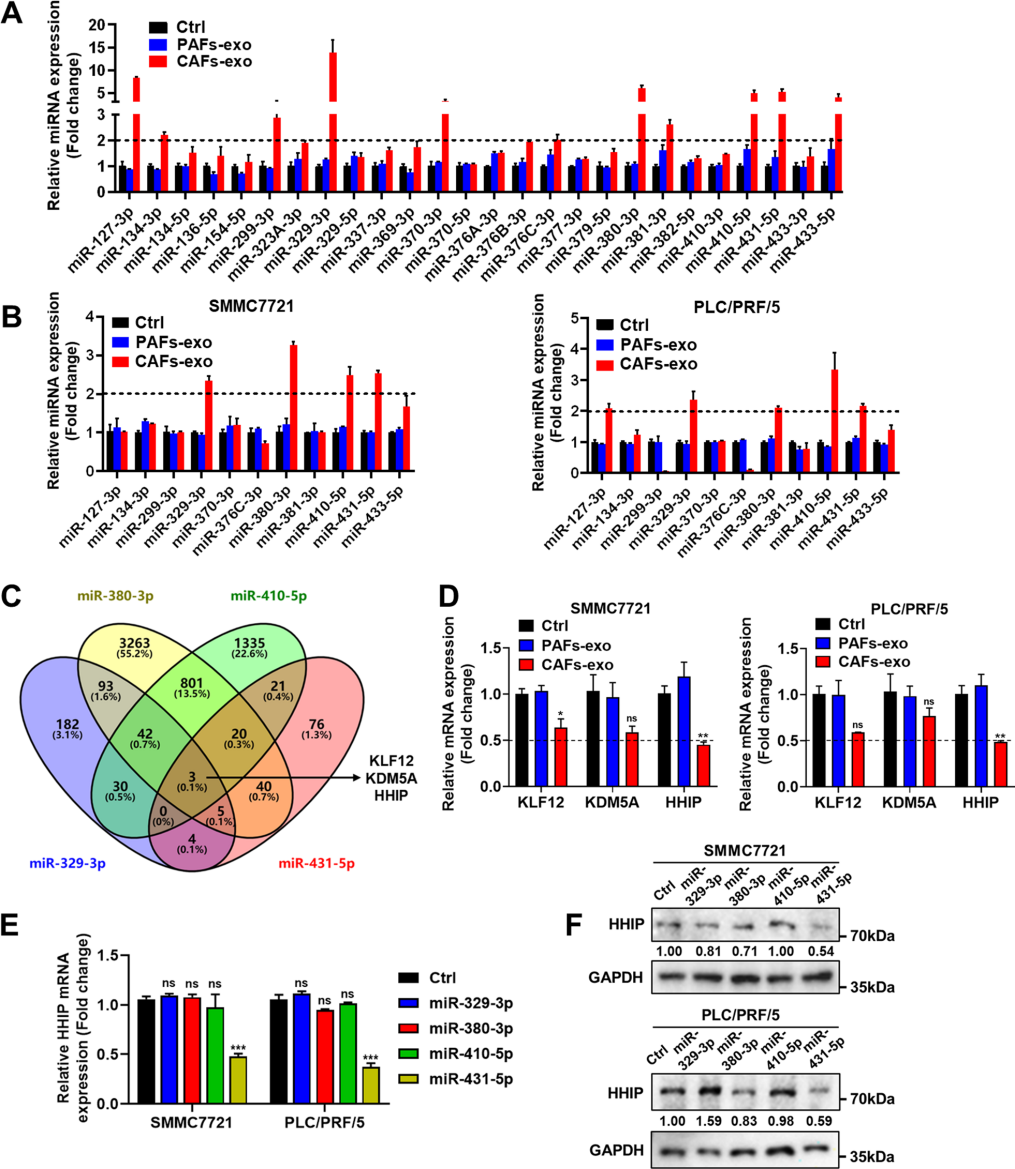
MicroRNAs (miRNAs) in the DLK1-DIO3 imprinted region derived from CAFs-exo inhibit HHIP expression in HCC cells. **A** The expression levels of miRNAs in the DLK1-DIO3 imprinted region derived from PAFs-exo and CAFs-exo were detected by RT-PCR assay. **B** The expression levels of miRNAs in the DLK1-DIO3 imprinted region in HCC cells co-cultured with PAFs-exo and CAFs-exo were detected by RT-PCR assay. **C** The target genes of miR-329-3p, miR-380-3p, miR-410-5p, and miR-431-5p were predicted using the TargetScan database. **D** The mRNA expression levels of KLF12, KDM5A, and HHIP in HCC cells were detected by RT-PCR assay. **E**, **F** The mRNA and protein expression levels of HHIP in HCC cells transfected with 4 miRNA mimics were detected by RT-PCR and WB assays

Luk et al. found that 21 miRNAs in the DLK1-DIO3 miRNA cluster were coordinately upregulated in a subset of HCC tissues, and HCC patients with overexpression of these miRNAs showed significantly poorer overall survival (OS) [26]. To investigate the potential molecules responsible for the role of CAFs-exo in promoting HCC progression, the expression levels of these miRNAs were detected by RT-PCR assay. The results demonstrated that 11 miRNAs were highly expressed in CAFs-exo compared with PAFs-exo (Fig. 4A). To identify the specific miRNAs that were transferred to HCC cells from CAFs-exo, we analyzed the expression levels of these miRNAs in HCC cells. The results showed that miR-329-3p, miR-380-3p, miR-410-5p, and miR-431-5p were upregulated in PLC/PRF/5 and SMMC7721 cells treated with CAFs-exo (Fig. 4B).

Kruppel like factor 12 (KLF12), lysine demethylase 5A (KDM5A), and HHIP were predicted to be target genes of miR-329-3p, miR-380-3p, miR-410-5p, and miR-431-5p using the TargetScan database (Fig. 4C). Because it was the most downregulated gene in the RT-PCR results, HHIP was selected for further investigations (Fig. 4D). We detected the expression level of HHIP in HCC cells treated with 4 miRNA mimics. The results showed that HHIP expression was decreased in HCC cells treated with miR-431-5p mimic at both the mRNA and protein level, indicating that HHIP was a potential target gene of miR-431-5p in HCC (Fig. 4E, F; Additional file 1: Fig. S4).

### HHIP is significantly downregulated in HCC tissues

**Fig. 5:**
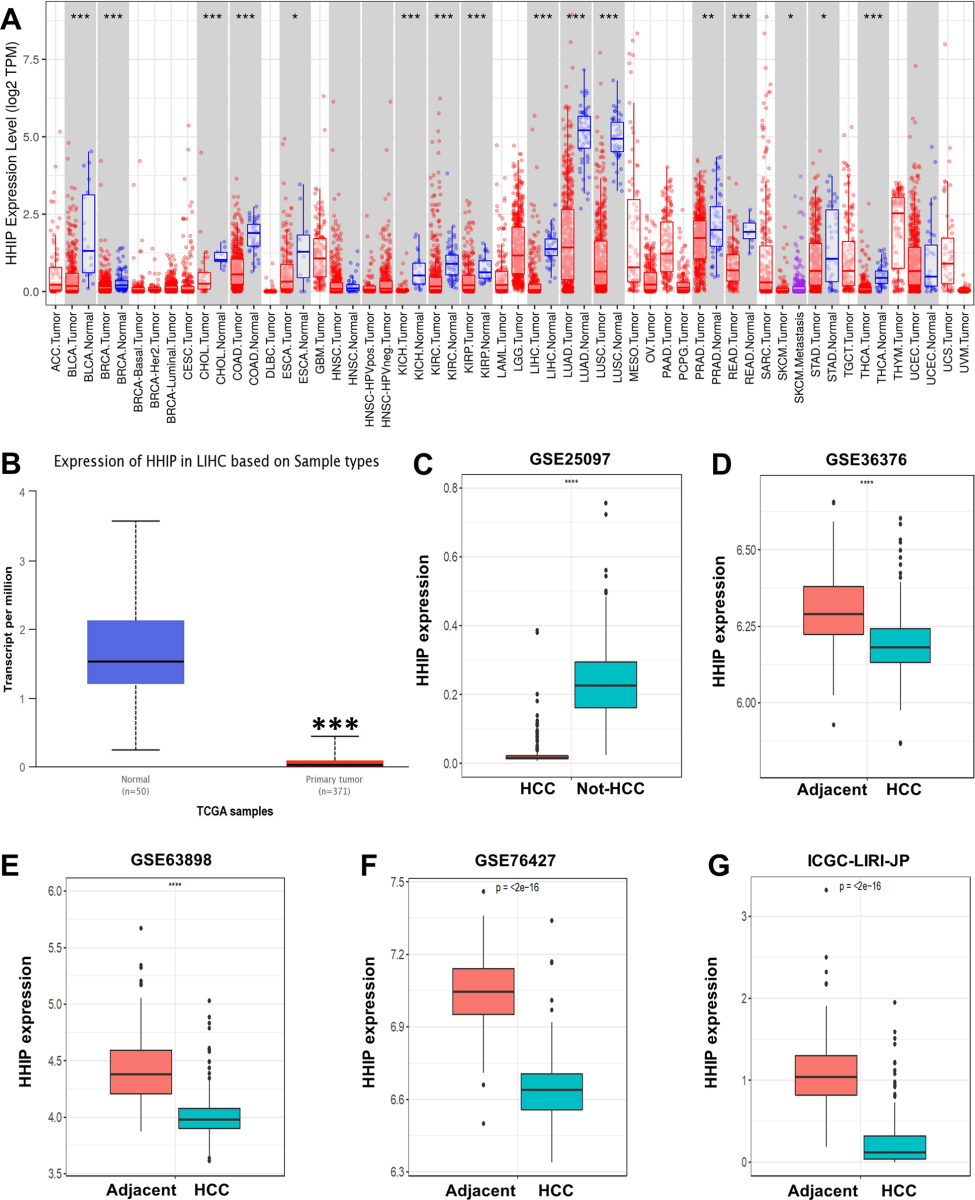
HHIP expression level in HCC. **A** The mRNA expression level of HHIP in tumor tissues and normal tissues was performed using the TIMER database. **B** The mRNA expression level of HHIP in The Cancer Genome Atlas-liver hepatocellular carcinoma (TCGA-LIHC) cohort. **C**–**F** The mRNA expression level of HHIP in HCC cohorts from the Gene Expression Omnibus (GEO) database. **G** The mRNA expression level of HHIP in the International Cancer Genome Consortium-Liver Cancer-RIKEN, JP (ICGC-LIRI-JP) cohort

The TIMER database was used to explore the expression level of HHIP in human cancers, which showed that HHIP was downregulated in a variety of tumor tissues (Fig. 5A). The analysis of the differential expression of HHIP in HCC cohorts from the TCGA database, GEO database, and ICGC database also confirmed that HHIP expression was decreased in HCC tissues (Fig. 5B–G).

**Fig. 6:**
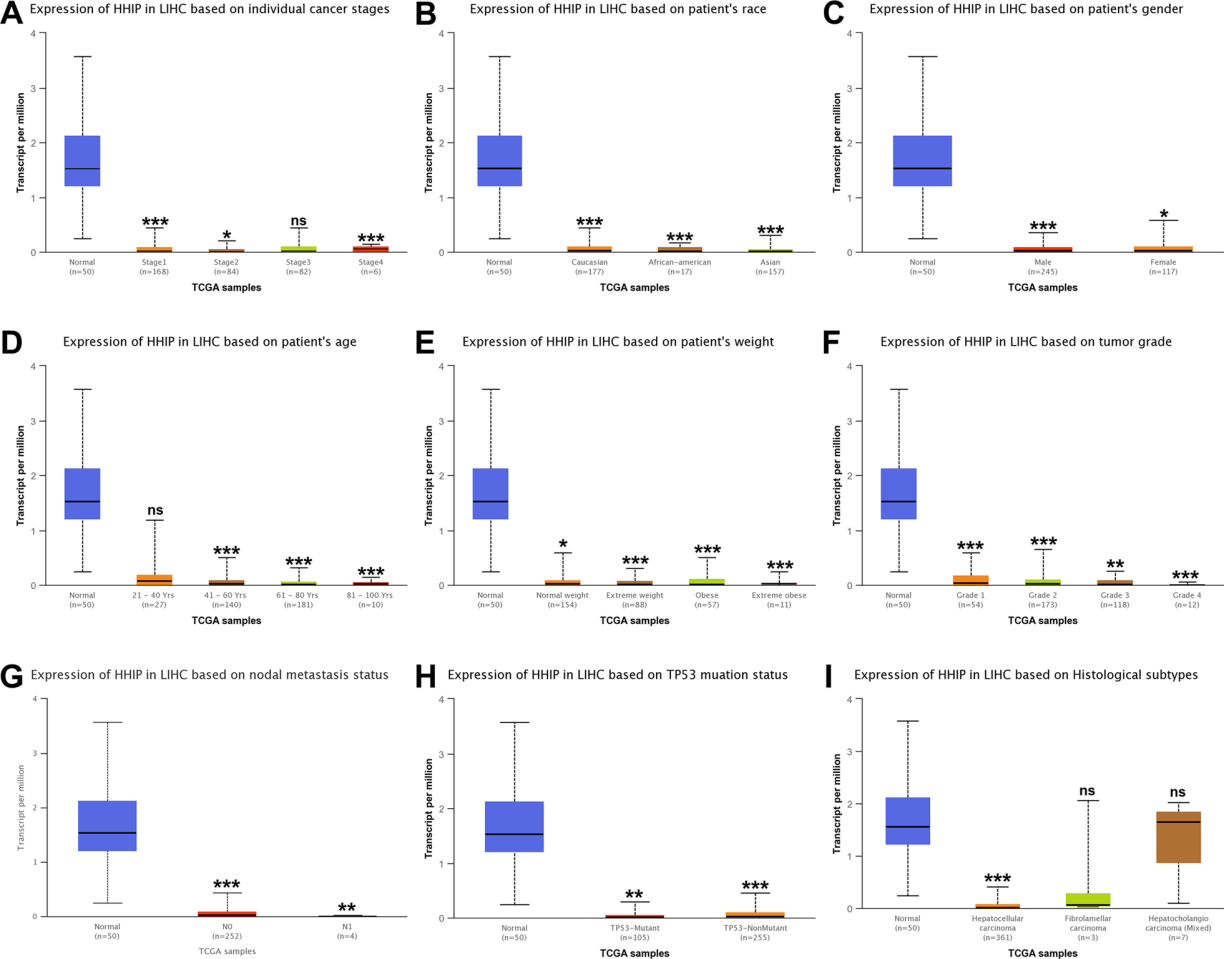
Differential expression of HHIP in the TCGA-LIHC subgroups. HHIP mRNA expression analysis in the TCGA-LIHC subgroups based on **A** individual cancer stages, **B** patients’ race, **C** patients’ gender, **D** patients’ age, **E** patients’ weight, **F** tumor grade, **G** nodal metastasis status, **H** TP53 mutation status, and **I** histological subtypes

We further explored HHIP expression level in HCC subgroups compared with normal tissues based on several clinical characteristics (individual cancer stages, race, gender, age, weight, tumor grade, nodal metastasis status, TP53 mutation status, histological subtypes) using the UALCAN database. HHIP downregulation was observed in different subgroups of HCC (Fig. 6A–I; Additional file 2: Table S1), implying that HHIP was a potential prognostic biomarker for HCC patients.

### Prognostic value of HHIP expression in HCC

**Fig. 7:**
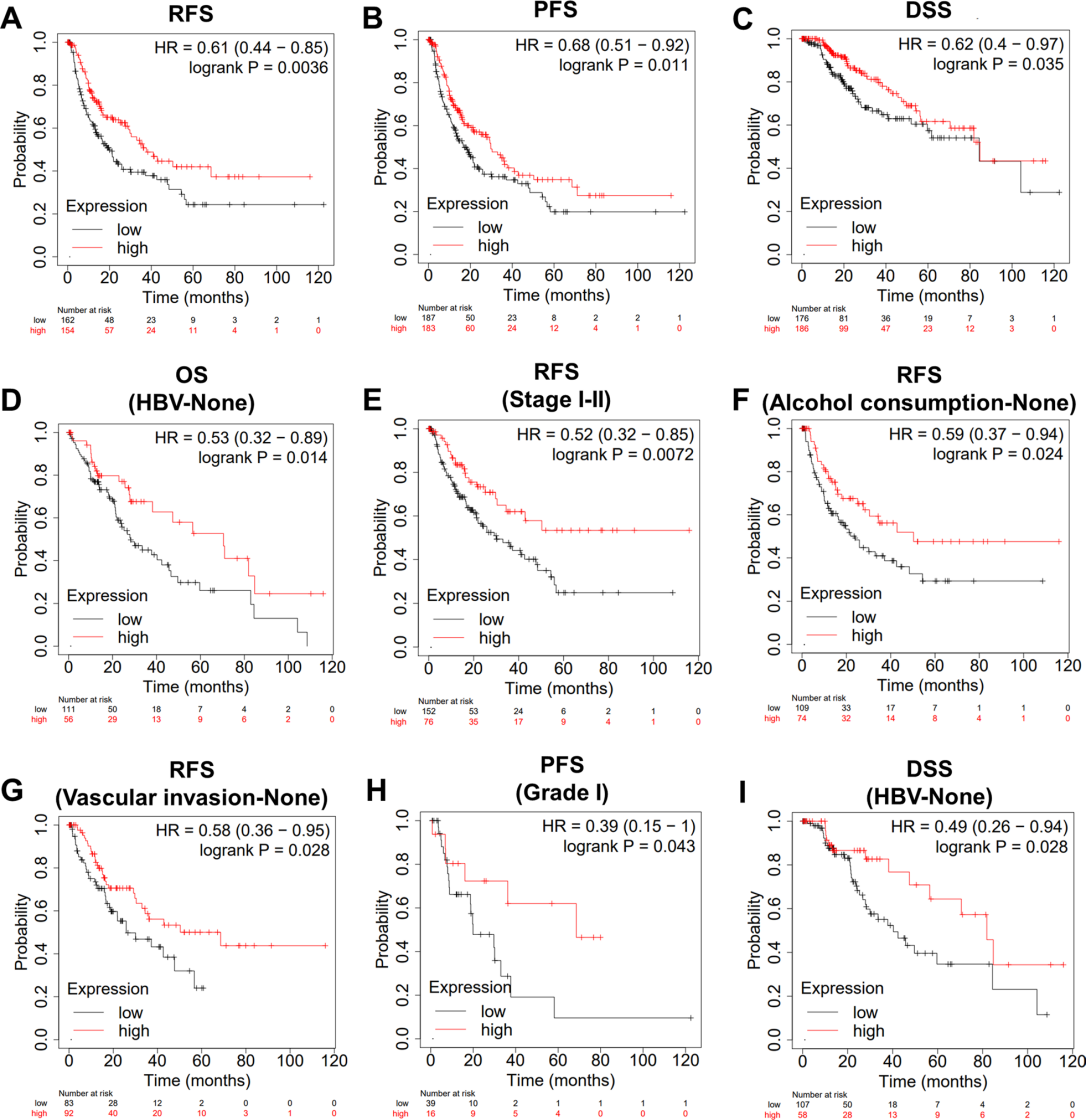
Prognostic analysis of HHIP expression in the TCGA-LIHC cohort using the Kaplan–Meier (KM) plotter database. **A**–**C** KM survival curves of relapse-free survival (RFS), progression-free survival (PFS), and disease-specific survival (DSS) of HCC patients according to HHIP expression level. **D** KM survival curves of overall survival (OS) of HCC patients in the HBV-None subgroup. **E**–**G** KM survival curves of RFS of HCC patients in the stage I-II subgroup, alcohol consumption-None subgroup, and vascular invasion-None subgroup, respectively. **H** KM survival curves of PFS of HCC patients in the grade I subgroup. **I** KM survival curves of DSS of HCC patients in the HBV-None subgroup

KM survival curves were used to assess the prognostic value of HHIP in the TCGA-LIHC cohort using the KM plotter database. The results showed that HCC patients with low HHIP expression level had shorter relapse-free survival (RFS), progression-free survival (PFS), and disease-specific survival (DSS) (Fig. 7A–C), indicating that low HHIP expression was associated with a poor outcome in HCC. In addition, we explored the prognostic significance of HHIP expression in the low recurrent risk subgroups of HCC. We found that HCC patients with low HHIP expression level had shorter OS in the HBV-None group, shorter RFS in the stage I-II group, alcohol consumption-None group, and vascular invasion-None group, shorter PFS in the grade I group, and shorter DSS in the HBV-None group (Fig. 7D–I).

### GSEA analysis of HHIP-related co-expressed genes in HCC

**Fig. 8:**
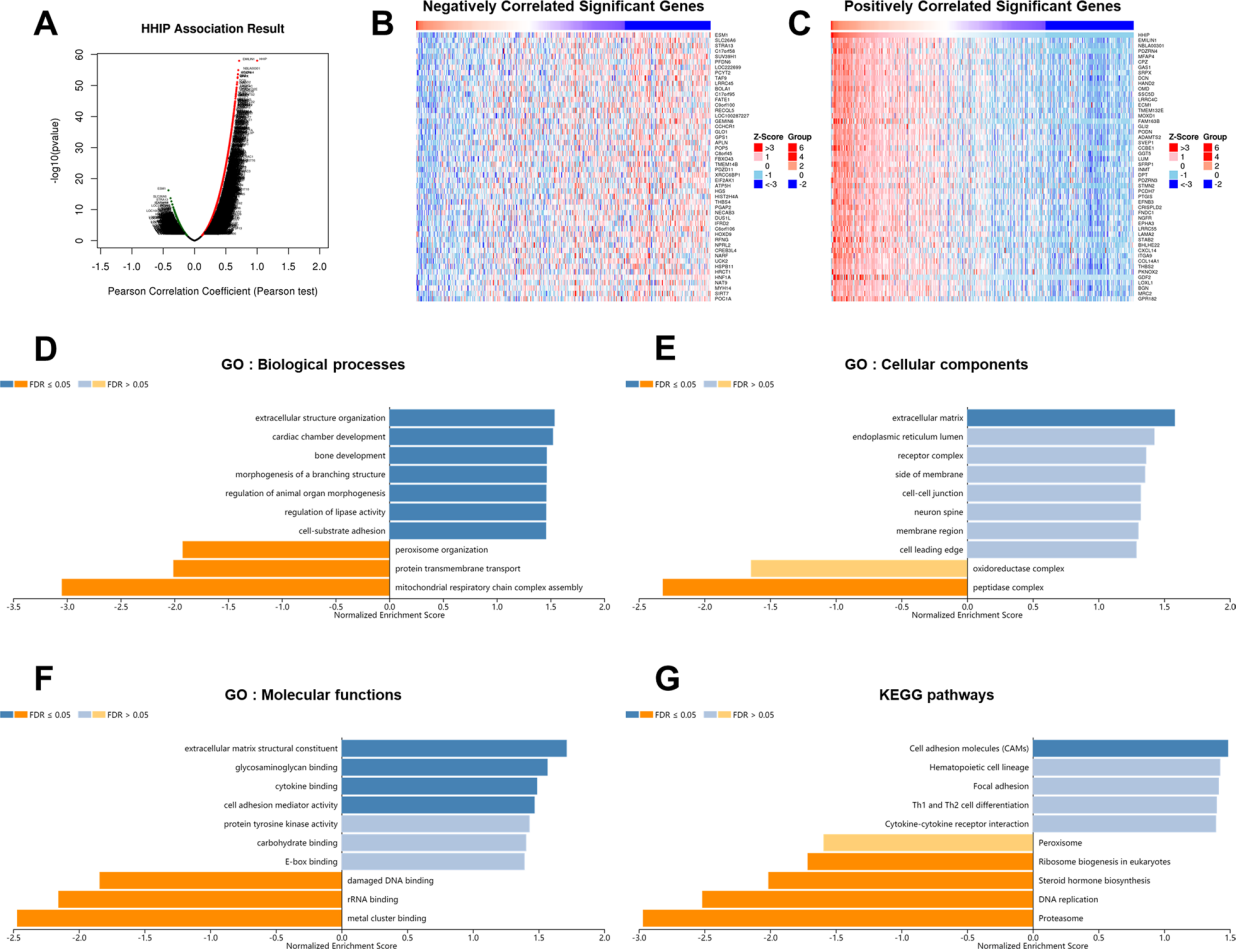
Enrichment analysis of HHIP-related co-expressed genes in the TCGA-LIHC cohort. **A** Analysis of HHIP-related co-expressed genes was performed using the LinkedOmics database. **B**, **C** The top 50 genes that were positively and negatively correlated with HHIP expression level were shown in heat maps. **D**–**G** Analysis of the significantly enriched Gene Ontology (GO) terms and Kyoto Encyclopedia of Genes and Genomes (KEGG) pathways (https://www.kegg.jp/kegg/) of HHIP-related co-expressed genes was performed using the Gene set enrichment analysis (GSEA) tool in the LinkedOmics database

To explore the underlying mechanisms of HHIP in HCC progression, the LinkedOmics database was used to identify the HHIP-related co-expressed genes in the TCGA-LIHC cohort. Overall, 11,748 genes were positively related to HHIP expression, while 8,174 genes were negatively related to HHIP expression (Fig. 8A). The top 50 HHIP-related co-expressed genes were shown in a heat map (Fig. 8B, C). GSEA analysis was used to analyze the GO terms and KEGG pathways of HHIP-related co-expressed genes. GO analysis showed that HHIP-related co-expressed genes were involved in cell-substrate adhesion and cell adhesion mediator activity (Fig. 8D–F; Additional file 2: Table S2), while KEGG analysis demonstrated that HHIP-related co-expressed genes were mainly associated with cell adhesion molecules (Fig. 8G; Additional file 2: Table S2).

## Discussion

HCC is one of the most prevalent malignancies worldwide with high rates of recurrence and metastasis [27]. There is an urgent need to understand the mechanisms of HCC progression to help develop novel therapeutic strategies for HCC. Here, we found that CAFs-exo could be internalized by HCC cells, and promoted cell proliferation, migration, and invasion. Further investigations revealed that miRNAs in the DLK1-DIO3 imprinted region were transferred from CAFs-exo to HCC cells, inhibiting the expression of HHIP in these cells. We then analyzed data from TCGA database, and found that HHIP expression was decreased in HCC tissues, moreover, it was positively correlated with OS of HCC patients. Functional enrichment analysis suggested that the function of HHIP was likely dependent on the regulation of cell adhesion in HCC. These findings help elucidate the communication details between CAFs-exo and HCC cells, and indicate that HHIP is a promising prognostic biomarker in HCC patients.

Exosomes secreted by tumor cells can modulate various biological processes in tumor progression, including tumor angiogenesis, metastasis, and immune escape [28]. Our results demonstrated that CAFs-exo contributed to the aggressive phenotype of HCC cells in vitro. These findings were in accordance with a previous report that showed that CAFs-exo could enhance EMT and cell stemness, leading to metastasis and chemotherapy resistance in colorectal cancer [29]. Increasing evidences had shown that miRNAs derived from tumor cells could be encapsulated in exosomes, and delivered to recipient cells to exert cancer-supporting functions by binding to sequences in the 3’-UTR of target mRNAs [30]. The DLK1-DIO3 miRNA cluster plays a significant role in cancer self-renewal and maintenance of the aggressive phenotype [31]. MiR-380-3p was upregulated in bladder cancer tissues, and promoted cell proliferation and mitochondrial respiration [32]. MiR-410-5p could promote tumor growth through degradation of miR-410-3p, which prevented miR-410-3p-mediated suppression of tumor angiogenesis [33]. Overexpression of miR-431-5p could promote colony formation and inhibited apoptosis in cervical adenocarcinoma [34]. However, the role and specific mechanisms of the DLK1-DIO3 miRNA cluster in HCC progression remain unclear.

KLF12 is a member of kruppel-like factor family, and its tumor suppressive and oncogenic functions in human cancers are increasingly appreciated [35]. KDM5A belongs to the KDM5 Jumonji histone demethylase subfamily, and is responsible for driving cell growth, differentiation, multi-drug resistance, invasion, and metastasis [36]. HHIP binds to all Hh ligands, and acts as a negative regulator of the Hh/GLI signaling pathway, which is abnormally activated in colorectal cancer, prostate cancer, and other cancers [37]. In this study, we found that low HHIP expression level could predict worse prognosis in HCC patients. Moreover, the prognostic value of HHIP was noted in HCC patients without HBV infection and alcohol consumption. The prognostic significance of HHIP in these low recurrent risk subgroups can help clinicians identify patients at high risk of recurrence and implement appropriate postoperative adjuvant treatments. However, the clinical significance of HHIP expression in HCC requires further investigation.

Previous studies reported that HHIP overexpression could inhibit cell proliferation, migration, and invasion in HCC [38], gastric cancer [39], and lung cancer [40]. A pervious study reported that DNA hypermethylation and/or loss of heterozygosity resulted in the downregulation of HHIP transcription, and Hh signal activation through the inactivation of HHIP was implicated in the pathogenesis of HCC [41]. Our data showed that HHIP-related co-expressed genes were closely associated with cell adhesion, indicating that HHIP might play an important role in HCC progression through regulating cell adhesion molecules.

Our study has several limitations. The results showed that miRNAs in the DLK1-DIO3 imprinted region were transferred from CAFs-exo to HCC cells, and promoted HCC progression by targeting HHIP. However, the role and mechanisms of HHIP in HCC progression need further clarification. Additionally, we found that HHIP expression level was decreased in HCC tissues, and low HHIP expression could predict poor prognosis in the TCGA-LIHC cohort. However, it should be noted that the potential prognostic value of HHIP requires additional validation in more HCC cohorts.

## Conclusion

In this study, we demonstrate that CAFs-exo are internalized by HCC cells, and can promote the aggressive phenotype of HCC cells. The expression levels of miR-329-3p, miR-380-3p, miR-410-5p, and miR-431-5p are upregulated in HCC cells treated with CAFs-exo compared with PAFs-exo, while HHIP expression level is significantly decreased. HHIP is a potential target gene of miR-431-5p in HCC. Bioinformatics analysis suggests that the function of HHIP in HCC may depend on the regulation of cell adhesion molecules. HHIP is expected to be a promising prognostic biomarker for HCC patients.

## Supplementary Information

**Additional file 1:** Supplementary figures.

**Additional file 2:** Supplementary tables.

## Data Availability

The datasets analyzed for this study could be found in The Cancer Genome Atlas database (https://portal.gdc.cancer.gov), Gene Expression Omnibus database (https://www.ncbi.nlm.nih.gov/), and International Cancer Genome Consortium data portal (https://dcc.icgc.org/). All data generated or analyzed during this study are included in this published article [and its supplementary information files].
