# Supplementary material for: Exosomal microRNAs in the DLK1-DIO3 imprinted region derived from cancer-associated fibroblasts promote progression of hepatocellular carcinoma by targeting hedgehog interacting protein
Source: BMC Gastroenterol. 2022 Dec 8;22:505. doi: 10.1186/s12876-022-02594-2 (PMC9730585; doi:10.1186/s12876-022-02594-2)
Supplement: Supplementary file 1 — Additional file 1: Supplementary figures. [file 12876_2022_2594_MOESM1_ESM.pdf]

Supplementary figure 1

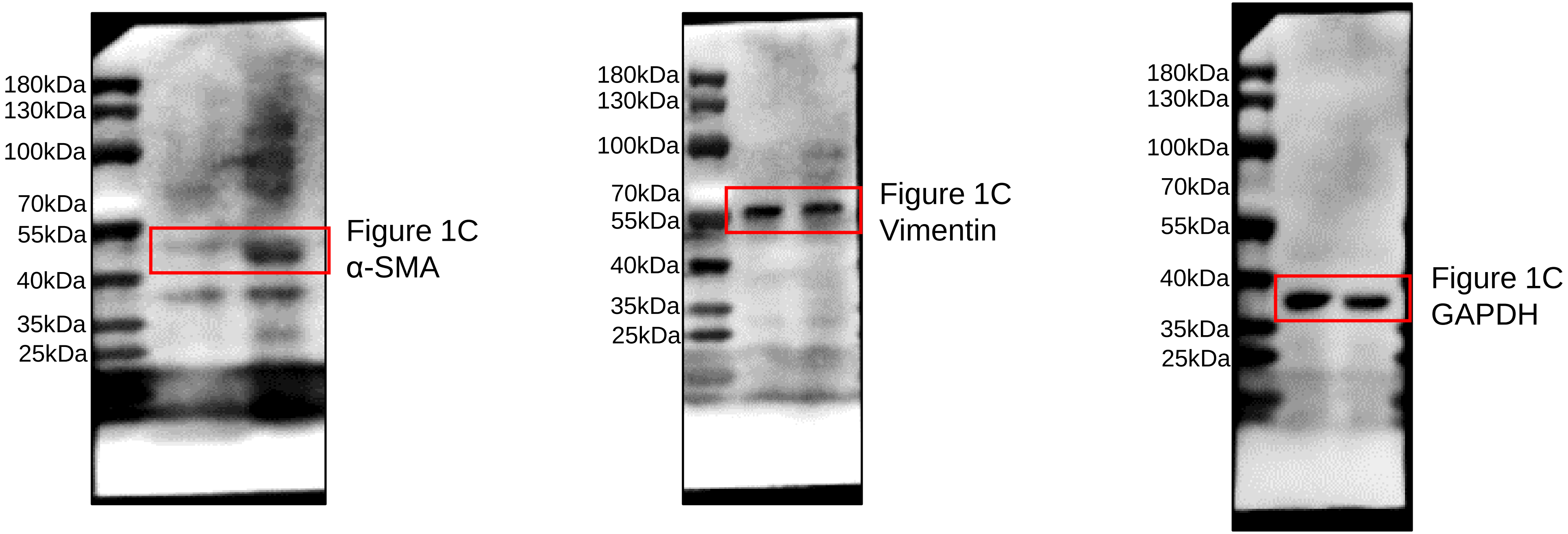

Supplementary figure 2

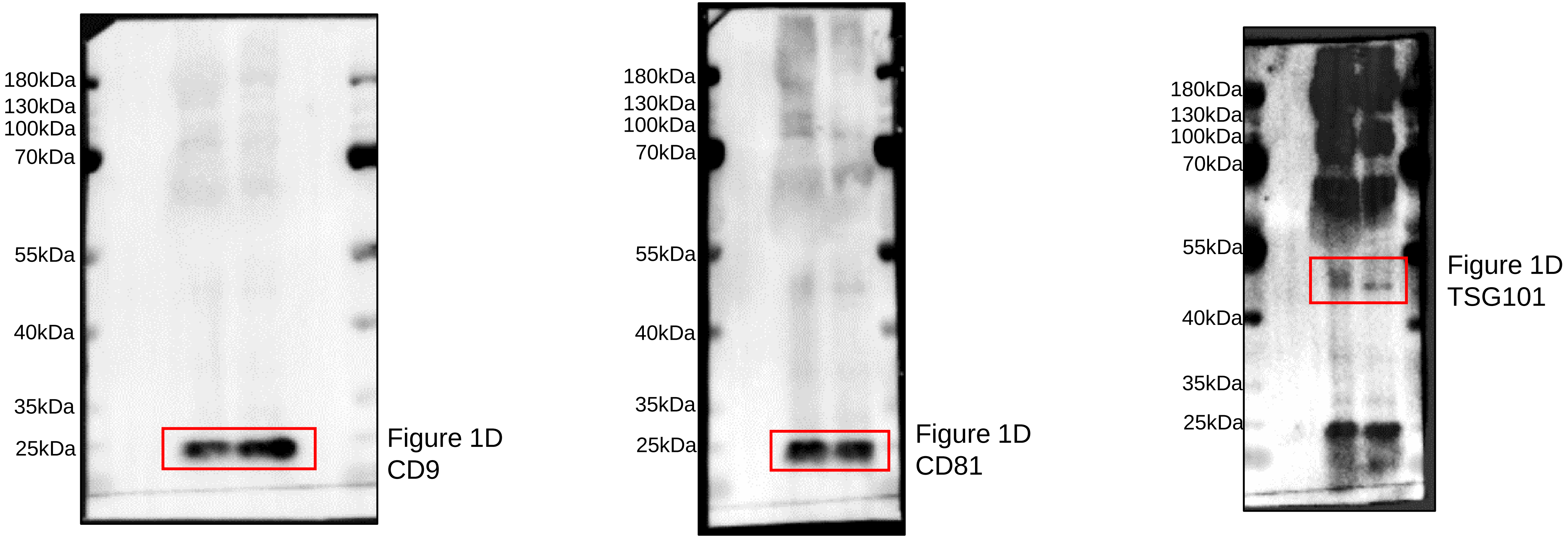

Supplementary figure 3

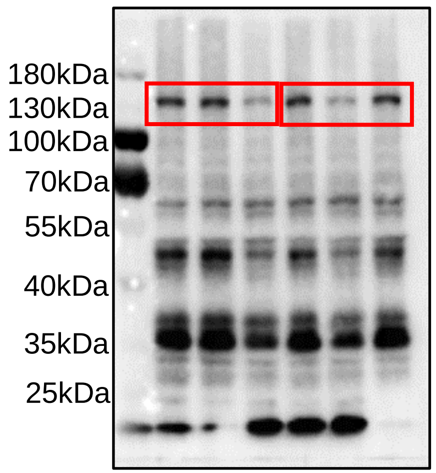

Figure 2D/3D
E-cadherin
SMMC7721

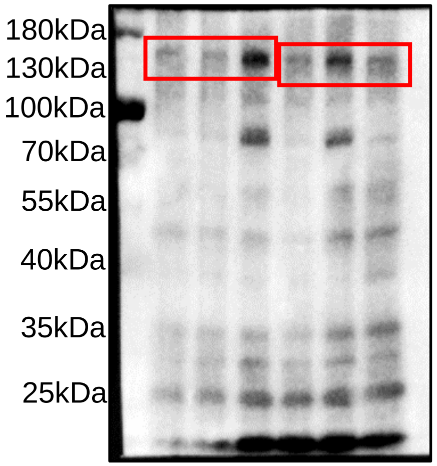

Figure 2D/3D
N-cadherin
SMMC7721

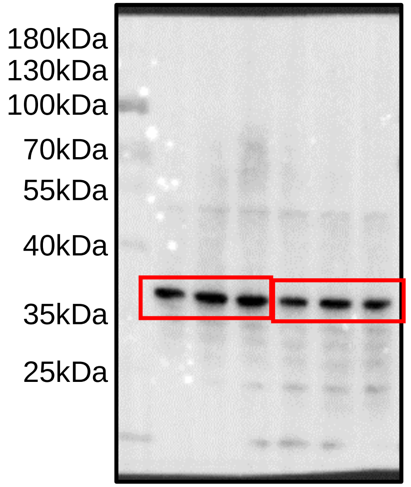

Figure 2D/3D
GAPDH
SMMC7721

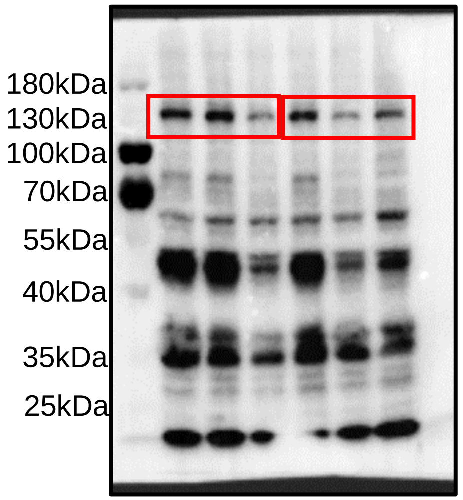

Figure 2D/3D
E-cadherin
PLC/PRF/5

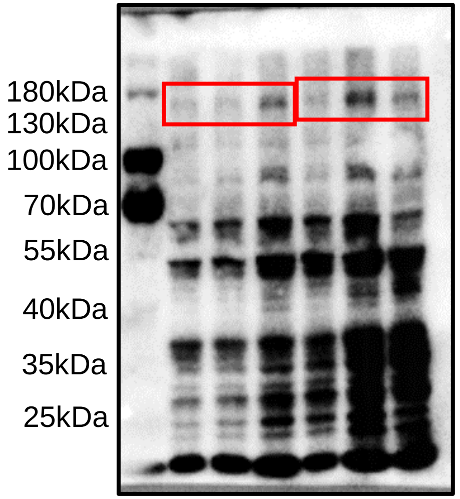

Figure 2D/3D
N-cadherin
PLC/PRF/5

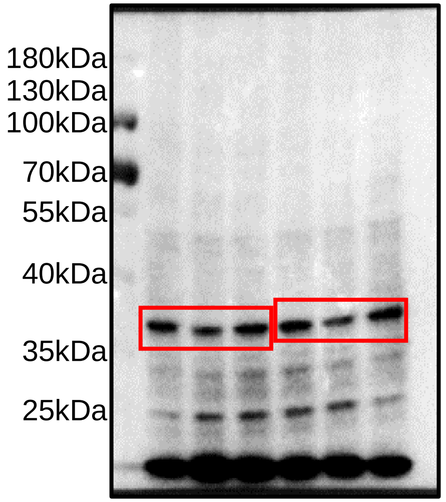

Figure 2D/3D
GAPDH
PLC/PRF/5

Supplementary figure 4

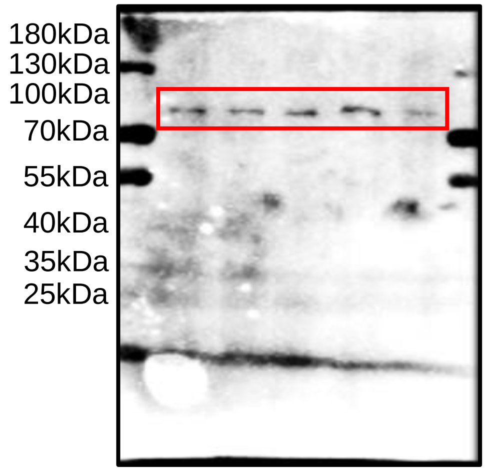

Figure 4F
HHIP
SMMC7721

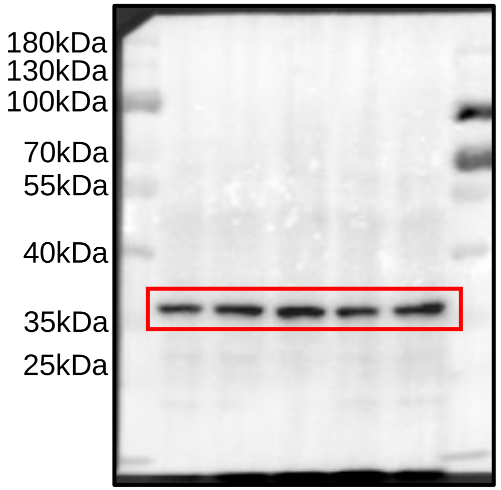

Figure 4F
GAPDH
SMMC7721

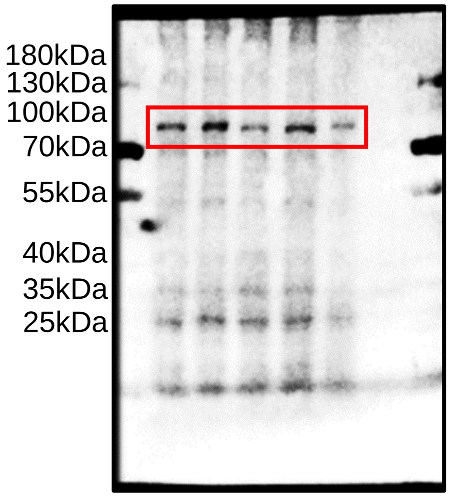

Figure 4F
HHIP
PLC/PRF/5

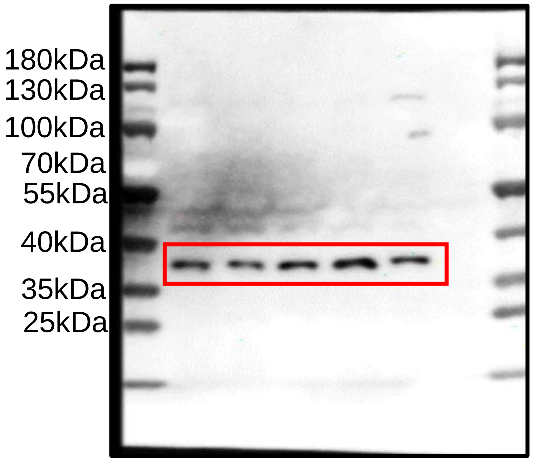

Figure 4F
GAPDH
PLC/PRF/5
