# Supplementary material for: Exosomal microRNAs in the DLK1-DIO3 imprinted region derived from cancer-associated fibroblasts promote progression of hepatocellular carcinoma by targeting hedgehog interacting protein
Source: BMC Gastroenterol. 2022 Dec 8;22:505. doi: 10.1186/s12876-022-02594-2 (PMC9730585; doi:10.1186/s12876-022-02594-2)
Supplement: Supplementary file 2 — Additional file 2: Supplementary tables. [file 12876_2022_2594_MOESM2_ESM.docx]

| **Supplementary Table 1. The HHIP mRNA expression for HCC based on different clinical characteristics by UALCAN.** | | | | |
| --- | --- | --- | --- | --- |
| **Variables** | **Different stages** | **N** | **Comparisons** | **Statistical significance** |
| **Sample types** | Normal | 50 | Normal vs. primary tumor | 5.16320000087944E-08 |
|  | Primary tumor | 371 |  |  |
| **Individual cancer stages** | Stage 1 | 168 | Normal vs. Stage 1 | 1.62436730732907E-12 |
|  | Stage 2 | 84 | Normal vs. Stage 2 | 0.0159157 |
|  | Stage 3 | 82 | Normal vs. Stage 3 | 0.26282 |
|  | Stage 4 | 6 | Normal vs. Stage 4 | 1.11022302462516E-16 |
| **Patients' race** | Caucasian | 177 | Normal vs. Caucasian | 0.00035746 |
|  | African-American | 17 | Norma vs. African-American | 1.64013247427874E-12 |
|  | Asian | 157 | Normal vs. Asian | 0.00003583000000007 |
| **Patients' gender** | Male | 245 | Normal vs. Male | 2.18509998983762E-09 |
|  | Female | 117 | Normal vs. Female | 0.028124 |
| **Patients' age** | 21-40 yrs | 27 | Normal vs. 21-40 yrs | 0.79086 |
|  | 41-60 yrs | 140 | Normal vs. 41-60 yrs | 0.00059908 |
|  | 61-80 yrs | 181 | Normal vs. 61-80 yrs | 1.62836411021772E-12 |
|  | 81-100 yrs | 10 | Normal vs. 81-100 yrs | 3.44169137633799E-15 |
| **Patients' weight** | Normal weight | 154 | Normal vs. Normal weight | 0.036996 |
|  | Extreme weight | 88 | Normal vs. Extreme weight | 8.88178419700125E-16 |
|  | Obese | 57 | Normal vs. Obese | 2.58681964737661E-13 |
|  | Extreme obese | 11 | Normal vs. Extreme obese | 0.0000166044999999615 |
| **Tumor grade** | Grade 1 | 54 | Normal vs. Grade 1 | 1.62836411021772E-12 |
|  | Grade 2 | 173 | Normal vs. Grade 2 | 0.00084072 |
|  | Grade 3 | 118 | Normal vs. Grade 3 | 0.00102159 |
|  | Grade 4 | 12 | Normal vs. Grade 4 | 2.87629999995431E-06 |
| **Nodal metastasis status** | N0 | 252 | Normal vs. N0 | 0.000115293 |
|  | N1 | 4 | Normal vs. N1 | 0.0098574 |
| **TP53 mutation status** | TP53-Mutant | 105 | Norma vs. TP53-Mutant | 0.0038621 |
|  | TP53-NonMutant | 255 | Normal vs. TP53-NonMutant | 1.89850000054115E-07 |
| **Tumor histology** | Hepatocellular carcinoma | 361 | Normal vs. Hepatocellular carcinoma | 4.05610000031587E-08 |
|  | Fibrolamellar carcinoma | 3 | Normal vs. Fibrolamellar carcinoma | 0.5368 |
|  | Hepatocholangio carcinoma (Mixed) | 7 | Normal vs. Hepatocholangio carcinoma (Mixed) | 0.3552 |

| **Supplementary Table 2. The top 10 terms of enrichment analysis of HHIP-related co-expressed genes by GSEA tool.** | | | | | | | |
| --- | --- | --- | --- | --- | --- | --- | --- |
| **Gene Set** | **Description** | **Size** | **Leading Edge Number** | **ES** | **NES** | **P** | **FDR** |
| **GO: Biological Process** | | | | | | | |
| GO:0071806 | Protein transmembrane transport | 59 | 29 | -0.47928 | -2.0099 | 0 | 0 |
| GO:0033108 | Mitochondrial respiratory chain complex assembly | 68 | 43 | -0.71153 | -3.0479 | 0 | 0 |
| GO:0007031 | Peroxisome organization | 79 | 35 | -0.44823 | -1.9236 | 0 | 0.0084723 |
| GO:0003205 | Cardiac chamber development | 158 | 65 | 0.75869 | 1.5233 | 0 | 0.015075 |
| GO:0043062 | Extracellular structure organization | 382 | 140 | 0.76272 | 1.5381 | 0 | 0.016281 |
| GO:2000027 | Regulation of animal organ morphogenesis | 204 | 74 | 0.7324 | 1.4615 | 0 | 0.018584 |
| GO:0060191 | Regulation of lipase activity | 92 | 34 | 0.74368 | 1.4601 | 0 | 0.018733 |
| GO:0031589 | Cell-substrate adhesion | 315 | 135 | 0.7303 | 1.4588 | 0 | 0.018798 |
| GO:0001763 | Morphogenesis of a branching structure | 196 | 72 | 0.73086 | 1.4648 | 0 | 0.018814 |
| GO:0060348 | Bone development | 196 | 70 | 0.73786 | 1.4656 | 0 | 0.018925 |
| **GO: Cellular Component** | | | | | | | |
| GO:1905368 | Peptidase complex | 85 | 51 | -0.54963 | -2.3175 | 0 | 0 |
| GO:0031012 | Extracellular matrix | 474 | 196 | 0.78285 | 1.5816 | 0 | 0.0046618 |
| GO:0005788 | Endoplasmic reticulum lumen | 291 | 79 | 0.71087 | 1.4261 | 0 | 0.064748 |
| GO:0043235 | Receptor complex | 391 | 135 | 0.68169 | 1.3635 | 0 | 0.067769 |
| GO:0098552 | Side of membrane | 459 | 200 | 0.67025 | 1.3554 | 0 | 0.07369 |
| GO:1990204 | Oxidoreductase complex | 95 | 46 | -0.42296 | -1.646 | 0 | 0.097495 |
| GO:0044309 | Neuron spine | 156 | 64 | 0.66017 | 1.322 | 0 | 0.10216 |
| GO:0005911 | Cell-cell junction | 418 | 157 | 0.6566 | 1.3229 | 0 | 0.10546 |
| GO:0098589 | Membrane region | 310 | 129 | 0.64847 | 1.3043 | 0 | 0.11499 |
| GO:0031252 | Cell leading edge | 372 | 145 | 0.64103 | 1.2898 | 0 | 0.1177 |
| **GO: Molecular Function** | | | | | | | |
| GO:0005201 | Extracellular matrix structural constituent | 151 | 86 | 0.86543 | 1.7143 | 0 | 0 |
| GO:0051540 | Metal cluster binding | 59 | 25 | -0.51759 | -2.4706 | 0 | 0 |
| GO:0005539 | Glycosaminoglycan binding | 198 | 79 | 0.78221 | 1.5685 | 0 | 0.004593 |
| GO:0019843 | rRNA binding | 60 | 30 | -0.54367 | -2.1565 | 0 | 0.014417 |
| GO:0019955 | Cytokine binding | 119 | 62 | 0.74158 | 1.4893 | 0 | 0.018802 |
| GO:0003684 | Damaged DNA binding | 67 | 29 | -0.46423 | -1.8427 | 0 | 0.019222 |
| GO:0098631 | Cell adhesion mediator activity | 59 | 20 | 0.74361 | 1.4697 | 0 | 0.026895 |
| GO:0004713 | Protein tyrosine kinase activity | 174 | 61 | 0.71794 | 1.4317 | 0 | 0.053931 |
| GO:0030246 | Carbohydrate binding | 238 | 84 | 0.70184 | 1.4055 | 0 | 0.067485 |
| GO:0070888 | E-box binding | 47 | 18 | 0.71752 | 1.3932 | 0 | 0.077506 |
| **KEGG Pathway** | | | | | | | |
| hsa03030 | DNA replication | 36 | 27 | -0.75956 | -2.5171 | 0 | 0 |
| hsa03050 | Proteasome | 44 | 25 | -0.73736 | -2.9686 | 0 | 0 |
| hsa00140 | Steroid hormone biosynthesis | 57 | 25 | -0.50408 | -2.014 | 0 | 0.0016579 |
| hsa04514 | Cell adhesion molecules (CAMs) | 136 | 73 | 0.74983 | 1.487 | 0 | 0.028778 |
| hsa03008 | Ribosome biogenesis in eukaryotes | 70 | 25 | -0.3398 | -1.7149 | 0 | 0.031708 |
| hsa04640 | Hematopoietic cell lineage | 93 | 62 | 0.72069 | 1.427 | 0 | 0.052571 |
| hsa04510 | Focal adhesion | 194 | 70 | 0.70961 | 1.416 | 0 | 0.061026 |
| hsa04060 | Cytokine-cytokine receptor interaction | 274 | 127 | 0.696 | 1.3953 | 0 | 0.06627 |
| hsa04658 | Th1 and Th2 cell differentiation | 90 | 47 | 0.714 | 1.4008 | 0 | 0.068065 |
| hsa04146 | Peroxisome | 82 | 34 | -0.30875 | -1.5937 | 0 | 0.076178 |
| Abbreviations: ES, enrichment score; NES, normalized enrichment score; FDR, false discovery rate. | | | | | | | |
